# Sex-specific associations between obstructive sleep apnea and thyroid hormone sensitivity in euthyroid adults

**DOI:** 10.1186/s13293-025-00821-5

**Published:** 2026-01-08

**Authors:** Beini Zhou, Yixuan Wang, Yuhan Wang, Jingyi Zhang, Qingfeng Zhang, Ke Hu

**Affiliations:** Department of Respiratory and Critical Care Medicine, Renmin Hospital of Wuhan University, 238 Jiefang Street, 430060 Wuhan, China

**Keywords:** Obstructive sleep apnea, Intermittent hypoxia, Thyroid hormone sensitivity.

## Abstract

**Background:**

The relationship between obstructive sleep apnea (OSA) and thyroid hormone sensitivity remains unclear. Thyroid hormone sensitivity indices may reveal subclinical hypothalamic-pituitary-thyroid (HPT) axis dysregulation beyond conventional hormone levels.

**Methods:**

We analyzed 718 euthyroid adults who underwent overnight sleep monitoring, using thyroid‑stimulating hormone index (TSHI), thyroid feedback quantile-based index (TFQI), parametric thyroid feedback quantile-based index (PTFQI), thyrotroph T4 resistance index (TT4RI), thyrotroph T3 resistance index (TT3RI) and the ratio of free triiodothyronine to free thyroxine (FT3/FT4 ratio) to assess central and peripheral thyroid hormone sensitivity. Analysis of covariance assessed differences across OSA severity after adjusting for age and BMI. Multivariable linear regression examined associations between OSA severity and thyroid hormone sensitivity indices in sex-stratified models. Correlations between OSA-related parameters and thyroid hormones sensitivity indices were further explored using quadratic prediction plots.

**Results:**

Among females, OSA patients showed higher FT4 and significantly increased TFQI, PTFQI, TSHI, and TT4RI, but lower FT3/FT4 ratio compared with non-OSA. TFQI (P for trend = 0.0395) and TT4RI (P for trend = 0.0293) were positively correlated with increasing OSA severity. OSA was independently associated with elevated TFQI (β = 0.26, 95% CI 0.010–0.42, *P* = 0.001), PTFQI (β = 0.20, 95% CI 0.05–0.35, *P* = 0.011), TSHI (β = 0.24, 95% CI 0.03–0.44, *P* = 0.025), and TT4RI (β = 6.82, 95% CI 0.59–13.05, *P* = 0.033). apnea-hypopnea index (AHI), oxygen desaturation index (ODI) were significantly correlated with TT4RI (*P* = 0.034, 0.021, respectively). No significant associations were observed in males.

**Conclusions:**

OSA is associated with impaired central and peripheral thyroid hormone sensitivity in euthyroid females, but not in males.

**Supplementary Information:**

The online version contains supplementary material available at 10.1186/s13293-025-00821-5.

## Highlights

Thyroid hormone sensitivity indices reveal subtle endocrine alterations in OSA patients despite normal hormone levels.

In females, OSA was associated with elevated TFQI, PTFQI, TSHI, and TT4RI, and reduced FT3/FT4 ratio, indicating impaired thyroid hormone sensitivity despite normal hormone levels.

Females with OSA may have a higher risk of endocrine dysregulation, and more attention needs to be paid to thyroid and metabolic health in this group.

**Supplementary Information:**

The online version contains supplementary material available at 10.1186/s13293-025-00821-5.

## Abstract (plain language summary)

This study explored how the body’s sensitivity to thyroid hormones may be affected in people with obstructive sleep apnea (OSA), even when their thyroid hormone levels appear normal. We examined six indices that reflect how well the brain and body respond to thyroid hormones. These indices can reveal hidden problems in hormone regulation that standard blood tests might miss. By analyzing data from 718 adults, the study found a clear pattern in females: those with OSA showed signs of reduced thyroid hormone sensitivity. As the severity of OSA increased, several indices also increased, suggesting a disruption in how the body regulates thyroid hormones. These changes were linked to how often breathing stopped and how low oxygen levels dropped during sleep. Interestingly, none of these patterns were seen in males. These findings suggest that OSA may interfere with thyroid hormone regulation in females, even when hormone levels are technically within the normal range. Understanding this difference may help explain why females with OSA sometimes show metabolic or energy-related problems and highlights that more detailed hormone assessments may be useful for females with OSA.

**Supplementary Information:**

The online version contains supplementary material available at 10.1186/s13293-025-00821-5.

## Background

Obstructive sleep apnea (OSA) is a common sleep-related breathing disorder characterized by recurrent episodes of upper airway collapse, intermittent hypoxia, and sleep fragmentation [1, 2]. It is increasingly recognized as a systemic disease with broad metabolic and endocrine consequences, beyond its established associations with cardiovascular and neurocognitive morbidity [3–5]. Among the endocrine systems potentially affected, the hypothalamic-pituitary-thyroid (HPT) axis has attracted growing attention. Several studies have reported associations between OSA and thyroid dysfunction, including both hypothyroidism and hyperthyroidism, yet the direction and consistency of these findings remain controversial [6–8]. Some investigations suggest that OSA is linked to elevated thyroid-stimulating hormone (TSH) levels and subclinical hypothyroidism, while others have observed suppressed TSH or altered free thyroid hormone concentrations, implying a hyperthyroid tendency [9, 10]. These inconsistencies indicate that the relationship between OSA and thyroid function may not be explained solely by absolute hormone levels.

Recent advances highlight the importance of thyroid hormone sensitivity indices, which quantify central and peripheral responsiveness to thyroid hormones within the euthyroid range [11, 12]. Indices such as the Thyroid Feedback Quantile-based Index (TFQI), parametric thyroid feedback quantile-based index (PTFQI), TSH Index (TSHI), Thyrotroph T4 Resistance Index (TT4RI) and thyrotroph T3 Resistance Index (TT3RI) integrate multiple parameters of the HPT axis to reflect subtle alterations in feedback regulation [11]. These indices may uncover latent endocrine dysregulation in individuals with normal thyroid hormone levels, thereby offering a more nuanced understanding of thyroid physiology in disease states.

Given that many OSA patients present with normal thyroid hormone concentrations, it is plausible that OSA may influence thyroid hormone sensitivity before overt thyroid dysfunction becomes apparent [13, 14]. This raises the question of whether OSA contributes to latent endocrine vulnerability, particularly in subgroups stratified by sex and disease severity. To address this question, the present study investigates the associations between OSA and thyroid hormone sensitivity indices in euthyroid individuals, providing new insights into the endocrine consequences of OSA.

## Methods

This manuscript was reported in accordance with the STROBE (Strengthening the Reporting of Observational Studies in Epidemiology) guidelines (**Tabel S1**).

### Study population

This study enrolled 718 patients who underwent sleep monitoring at the Center for Respiratory and Sleep Medicine, Renmin Hospital of Wuhan University from June 2023 to June 2025. Inclusion criteria: age ≥ 18 years; serum levels of thyroid hormones-including free triiodothyronine (FT3), free thyroxine (FT4), and TSH, were within normal reference range. Exclusion criteria were as follows: history of thyroid disease; current use of antithyroid medications or thyroid hormone replacement therapy; history of OSA treatment, including continuous positive airway pressure or surgical intervention; use of anxiolytics, antidepressants, hypnotics, or antipsychotic medications; history of malignant tumor; and missing data. This study was approved by the Ethics Committee of Renmin Hospital of Wuhan University. As a retrospective study, informed consent was waived.

### Data collection

Patient data were extracted from electronic medical records, including demographic information, laboratory test results. Demographic data included sex, age, body mass index (BMI), smoking and drinking, menopausal status in female participants, and medical history such as hypertension, diabetes mellitus, and coronary heart disease (CHD). Information on continuous medication use, including lipid lowering drugs, antihypertensive agents, antidiabetic drugs, and antiplatelet drugs was collected. Laboratory parameters comprised serum TSH, FT3, and FT4 levels, measured from peripheral blood samples collected in the early morning under fasting conditions.

### Assessment of OSA

Each participant underwent overnight sleep monitoring using an ultra-wideband bio-radar sleep screening device. This portable, contactless system consisted of a radar transmitter (ZG-S01D) and a finger photoplethysmography sensor (ZG-P11F). It continuously tracked respiratory patterns and body movements, recorded oxygen saturation and heart rate, and identified respiratory events using proprietary algorithms [15]. All participants were monitored for at least 7 consecutive hours during nighttime sleep. A detailed report was generated the following morning, including the apnea-hypopnea index (AHI), oxygen desaturation index (ODI), percentage of time with oxygen saturation below 90% (T90), mean blood oxygen saturation (Mean SpO₂), minimum blood oxygen saturation (Mini SpO₂), mean apnea time, as well as sleep duration, sleep efficiency, and wake after sleep onset (WASO). The percentage of total sleep time (sec) spent with apnoea and hypopnoea time (sec) is denoted as apnea-hypopnea time (AHT%). AHT% was calculated as (AHI × Mean apnea time/3600) × 100% [16]. OSA severity was classified based on AHI as follows: non-OSA (AHI < 5), mild OSA (5 ≤ AHI < 15), moderate OSA (15 ≤ AHI < 30), severe OSA (AHI ≥ 30) [17].

### Calculation of thyroid hormone sensitivity indices

Thyroid hormone sensitivity was evaluated using the following indices: TSHI, TFQI, PTFQI, TT4RI, TT3RI, and FT3/FT4 ratio. TSHI, TFQI, PTFQI, TT4RI, and TT3RI reflect central thyroid hormone sensitivity, while the FT3/FT4 ratio indicates peripheral thyroid hormone sensitivity. The formulas used were: TSHI = ln(TSH, mU/L) + 0.1345 × FT4 (pmol/L) [18]; TFQI = CDF(FT4) − [1 − CDF(TSH)] [11]; PTFQI = Φ[(FT4 − µFT4)/σFT4] − {1 − Φ[(lnTSH − µlnTSH)/σlnTSH]} [19]; TT4RI = FT4 (pmol/L) × TSH (mIU/L); TT3RI = FT3 (pmol/L) × TSH (mIU/L); FT3/FT4 ratio = FT3 (pmol/L) / FT4 (pmol/L). Higher central thyroid hormone sensitivity indices suggest impaired central thyroid hormone sensitivity, while a lower FT3/FT4 ratio indicates reduced peripheral thyroid hormone sensitivity.

### Sample size calculation

The required sample size was estimated based on the main analytical approaches of the study, including one-way ANCOVA and multiple linear regression. For ANCOVA, Cohen’s *f* effect size method was applied. With α = 0.05, power = 0.80, and assuming a medium effect size (*f* = 0.25), the required total sample size was approximately 180. For multiple linear regression, the incremental explanatory power of the main exposure was set at ΔR^2^ = 0.08, corresponding to *f* ^2^ = 0.089-0.10 under a baseline model R^2^ = 0.10–0.20. With α = 0.05 and power = 0.80, the required sample size was approximately 112–125. We adopted the maximum value of 180 as the minimum overall sample size target. Ultimately, 718 patients (527 males and 191 females) were enrolled, which far exceeded the required minimum, thereby ensuring adequate statistical power.

### Statistical analysis

Continuous variables were expressed as medians with interquartile ranges (IQR), and categorical variables as counts and percentages. Comparisons between non-OSA and OSA groups were performed using the Wilcoxon rank sum test for continuous variables and Pearson’s Chi-squared test or Fisher’s exact test for categorical variables. Analysis of covariance (ANCOVA) was conducted to compare thyroid hormone sensitivity indices across OSA severity groups, adjusting for age and BMI. Linear regression models were used to assess the association between OSA and thyroid hormone sensitivity in male and female subgroups, adjusting for age, BMI, sleep duration, WASO, sleep efficiency, smoking, drinking, menopausal status (in females) and medical history. Further analyses explored the relationships between OSA-related parameters (AHI, ODI, T90, AHT%) and thyroid hormone sensitivity indices. These relationships were modeled using multiple linear regression and visualized through quadratic prediction plots, where the central line represents the model-predicted mean and the shaded area indicates the 95% confidence interval (CI). To reduce skewness and improve model fit, T90 was log-transformed prior to regression and curve fitting analyses. Analyses were performed using R software version 4.3.0 (http://www.r-project.org), and a two-sided *P* < 0.05 was considered as significant.

## Results

### Baseline characteristics stratified by sex and OSA status

A total of 718 subjects were included in the study, including 527 males and 191 females. In both sexes, individuals with OSA exhibited higher BMI and prevalence of diabetes mellitus. Among males, no significant differences in thyroid hormone levels or thyroid hormone sensitivity indices were observed between the OSA and non-OSA groups (Table 1). In contrast, females with OSA showed higher FT4 concentrations and significant alterations in thyroid hormone sensitivity indices, characterized by increased TFQI, PTFQI, TSHI, and TT4RI values and a lower FT3/FT4 ratio (*P* = 0.011) (Table 2).

**Table 1:** Baseline characteristics of male participants by OSA status

Characteristic	Overall *N* = 527^1^	Non-OSA *N* = 45^1^	OSA *N* = 482^1^	*p*-value^2^
Age, years	51 (39, 62)	52 (33, 64)	51 (39, 62)	0.656
BMI, kg/m^2^	27.13 (24.73, 29.41)	24.49 (23.66, 27.46)	27.34 (24.96, 29.75)	< 0.001
AHI, events/h	27.9 (12.7, 47.1)	3.2 (2.1, 3.8)	31.1 (16.0, 49.3)	< 0.001
Mean apnea time, s	23 (21, 25)	23 (21, 26)	23 (21, 25)	0.439
AHT, %	18 (8, 30)	2 (1, 3)	19 (10, 31)	< 0.001
Mean SpO2, %	94 (92.7, 95)	96 (95, 97)	94 (92.2, 95)	< 0.001
Mini SpO2, %	79 (69, 85)	90 (86, 91)	78 (67, 84)	< 0.001
ODI, times/h	24.3 (11.7, 42.0)	3.5 (2.1, 5.5)	26.6 (15.0, 46.5)	< 0.001
T90, %	5.8 (1.1, 17.5)	0.0 (0.0, 0.5)	6.9 (1.8, 19.2)	< 0.001
Sleep duration, min	325 (271, 366)	372 (334, 404)	317 (266, 360)	< 0.001
WASO, min	59 (35, 95)	34 (23, 44)	65 (39, 102)	< 0.001
Sleep efficiency, %	77.0 (68.4, 85.0)	85.8 (84.6, 87.8)	75.9 (67.5, 84.3)	< 0.001
FT3, pg/mL	3.54 (3.24, 3.79)	3.55 (3.25, 3.72)	3.53 (3.24, 3.79)	0.884
FT4, ng/dL	1.19 (1.11, 1.31)	1.18 (1.12, 1.28)	1.19 (1.11, 1.31)	0.802
TSH, µIU/mL	1.86 (1.32, 2.59)	1.93 (1.38, 2.26)	1.86 (1.32, 2.60)	0.663
Smoking, n (%)	299 (56.7%)	19 (42.2%)	280 (58.1%)	0.040
Drinking, n (%)	207 (39.3%)	9 (20.0%)	198 (41.1%)	0.006
Hypertension, n (%)	404 (77%)	37 (82.2%)	367 (76.5%)	0.380
Diabetes mellitus, n (%)	122 (23.2%)	5 (11.1%)	117 (24.4%)	0.044
CHD, n (%)	98 (18.7%)	10 (22.2%)	88 (18.4%)	0.526
Lipid lowering drugs, n (%)	99 (18.8%)	7 (15.6%)	92 (19.1%)	0.562
Antihypertensive agents, n (%)	282 (53.5%)	22 (48.9%)	260 (53.9%)	0.516
Antidiabetic drugs, n (%)	87 (16.5%)	6 (13.3%)	81 (16.8%)	0.549
Antiplatelet drugs, n (%)	72 (13.7%)	5 (11.1%)	67 (13.9%)	0.602
TFQI	0.02 (-0.22, 0.26)	0.01 (-0.12, 0.11)	0.03 (-0.24, 0.28)	0.632
TSHI	2.70 (2.35, 3.06)	2.69 (2.51, 2.83)	2.71 (2.35, 3.08)	0.618
TT4RI	28 (20, 40)	30 (22, 34)	28 (20, 40)	0.636
PTFQI	-0.11 (-0.39, 0.14)	-0.13 (-0.26, 0.02)	-0.10 (-0.41, 0.17)	0.527
TT3RI	10.0 (7.1, 13.7)	9.8 (7.6, 12.8)	10.0 (7.1, 13.9)	0.650
FT3/FT4 ratio	0.35 (0.32, 0.38)	0.35 (0.32, 0.39)	0.35 (0.32, 0.38)	0.689

^1^Median (Q1, Q3); n (%)

^2^Wilcoxon rank sum test; Pearson’s Chi-squared test

OSA, obstructive sleep apnea; BMI, body mass index; AHI, apnea hypopnea index; AHT, apnea hypopnea time; ODI, oxygen desaturation index; T90, the percentage of time with oxygen saturation less than 90%; WASO, wake after sleep onset; FT3, free triiodothyronine; FT4, free thyroxine; TSH, thyroid-stimulating hormone; CHD, coronary heart disease; TFQI, thyroid feedback quantile-based index; TSHI, TSH Index; TT4RI, thyrotroph T4 resistance index; PTFQI, parametric thyroid feedback quantile–based index; TT3RI, thyrotroph T3 resistance index

**Table 2:** Baseline characteristics of female participants by OSA status

Characteristic	Overall *N* = 191^1^	Non-OSA *N* = 29^1^	OSA *N* = 162^1^	*P*-value^2^
Age, years	57 (42, 65)	49 (39, 59)	58 (43, 66)	0.050
BMI, kg/m^2^	26.17 (22.89, 31.20)	23.19 (20.70, 26.17)	26.98 (23.71, 31.25)	0.001
AHI, events/h	19.0 (8.9, 35.3)	2.1 (1.3, 3.7)	25.6 (11.8, 38.5)	< 0.001
Mean apnea time, s	23 (21, 25)	25 (21, 29)	23 (21, 24)	0.002
AHT, %	12 (6, 22)	2 (1, 2)	16 (8, 24)	< 0.001
MeanSpO2, %	94 (93, 95.48)	96 (95, 97)	94 (93, 95)	< 0.001
MiniSpO2, %	81 (75, 86)	89 (88, 91)	79 (73, 84)	< 0.001
ODI, times/h	18.8 (10.4, 30.8)	4.5 (2.2, 7.0)	21.2 (14.1, 32.8)	< 0.001
T90, %	3.7 (0.7, 8.1)	0.1 (0.0, 0.4)	4.8 (1.8, 9.4)	< 0.001
Sleep duration, min	345 (308, 374)	370 (338, 387)	341 (302, 370)	0.012
WASO, min	52 (34, 89)	36 (24, 43)	59 (37, 92)	< 0.001
Sleep efficiency, %	80.2 (72.4, 85.9)	85.9 (85.2, 87.5)	78.3 (70.4, 84.3)	< 0.001
FT3, pg/mL	3.31 (3.07, 3.50)	3.27 (3.08, 3.45)	3.32 (3.06, 3.51)	0.557
FT4, ng/dL	1.14 (1.05, 1.25)	1.08 (0.97, 1.13)	1.16 (1.06, 1.28)	< 0.001
TSH, µIU/mL	2.29 (1.61, 2.96)	2.03 (1.66, 2.56)	2.33 (1.61, 3.04)	0.192
Smoking, n (%)	11 (5.8%)	1 (3.4%)	10 (6.2%)	1.00
Drinking, n (%)	5 (2.6%)	0 (0.0%)	5 (3.1%)	1.00
Menopausal status, n (%)	126 (66.0%)	14 (48.3%)	112 (69.1%)	0.029
Hypertension, n (%)	121 (63.4%)	12 (41.4%)	109 (67.3%)	0.008
Diabetes mellitus, n (%)	44 (23.0%)	2 (6.9%)	42 (25.9%)	0.025
CHD, n (%)	32 (16.8%)	1 (3.4%)	31 (19.1%)	0.054
Lipid lowering drugs, n (%)	30 (15.7%)	3 (10.3%)	27 (16.7%)	0.580
Antihypertensive agents, n (%)	88 (46.1%)	9 (31.0%)	79 (48.8%)	0.078
Antidiabetic drugs, n (%)	28 (14.7%)	1 (3.4%)	27 (16.7%)	0.085
Antiplatelet drugs, n (%)	26 (13.6%)	1 (3.4%)	25 (15.4%)	0.137
TFQI	0.01 (-0.24, 0.26)	-0.23 (-0.32, -0.13)	0.06 (-0.17, 0.31)	< 0.001
TSHI	2.79 (2.48, 3.09)	2.56 (2.47, 2.65)	2.86 (2.50, 3.13)	0.001
TT4RI	33 (25, 44)	28 (24, 32)	34 (25, 45)	0.013
PTFQI	-0.07 (-0.31, 0.14)	-0.28 (-0.37, -0.17)	-0.03 (-0.30, 0.17)	< 0.001
TT3RI	11.2 (8.1, 14.5)	10.1 (8.5, 12.9)	11.5 (8.1, 15.1)	0.222
FT3/FT4 ratio	0.34 (0.30, 0.39)	0.37 (0.33, 0.40)	0.34 (0.30, 0.38)	0.011

^1^Median (Q1, Q3); n (%)

^2^Wilcoxon rank sum test; Pearson’s Chi-squared test; Fisher’s exact test

OSA, obstructive sleep apnea; BMI, body mass index; AHI, apnea hypopnea index; AHT, apnea hypopnea time; ODI, oxygen desaturation index; T90, the percentage of time with oxygen saturation less than 90%; WASO, wake after sleep onset;FT3, free triiodothyronine; FT4, free thyroxine; TSH, thyroid-stimulating hormone; CHD, coronary heart disease; TFQI, thyroid feedback quantile-based index; TSHI, TSH Index; TT4RI, thyrotroph T4 resistance index; PTFQI, parametric thyroid feedback quantile–based index; TT3RI, thyrotroph T3 resistance index

### Sex-stratified associations between OSA severity and thyroid hormone sensitivity indices

**Fig. 1:**
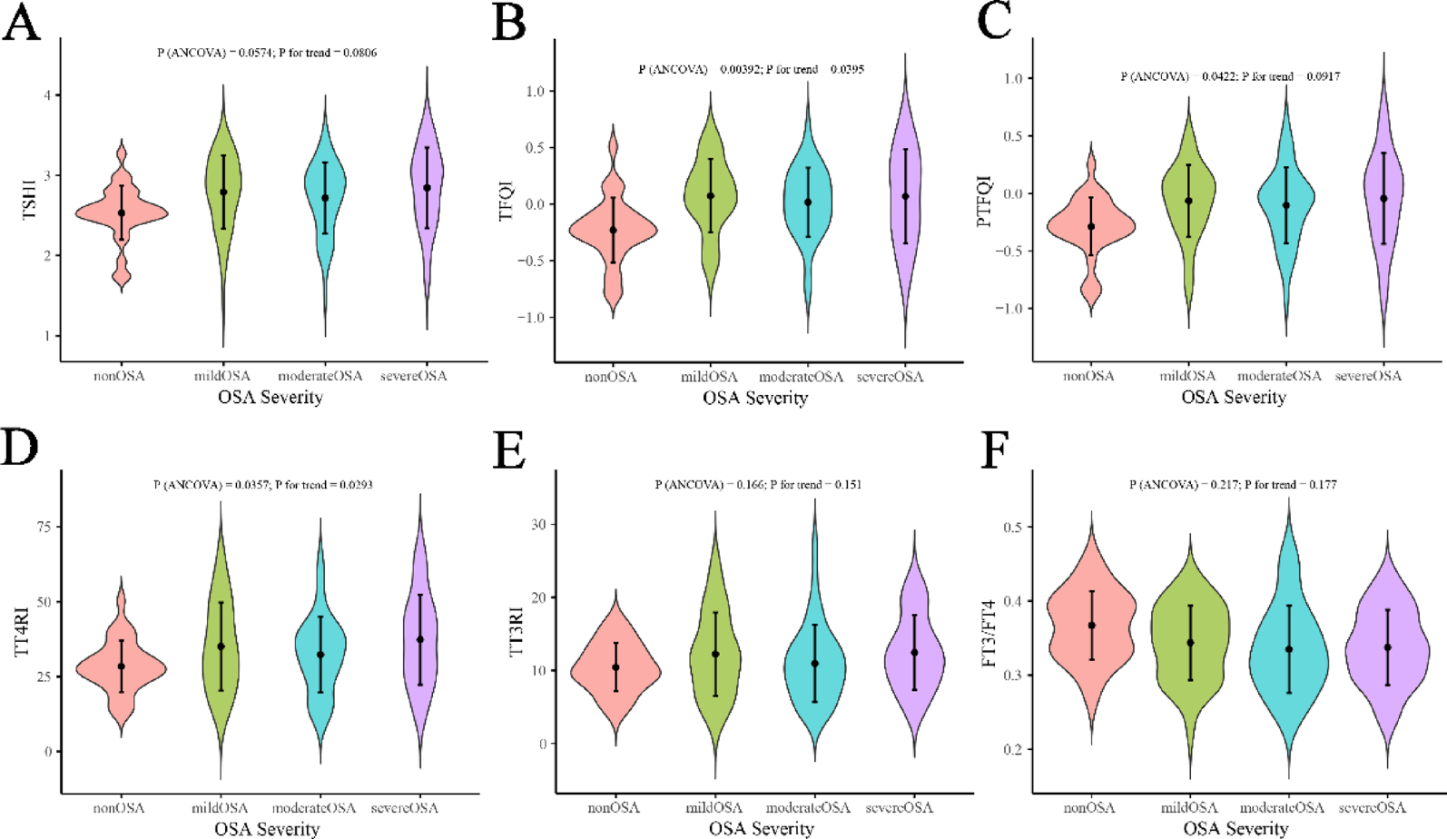
ANCOVA-based distribution of thyroid hormone sensitivity indices across OSA severity in females. ANCOVA, analysis of covariance; OSA, obstructive sleep apnea

**Fig. 2:**
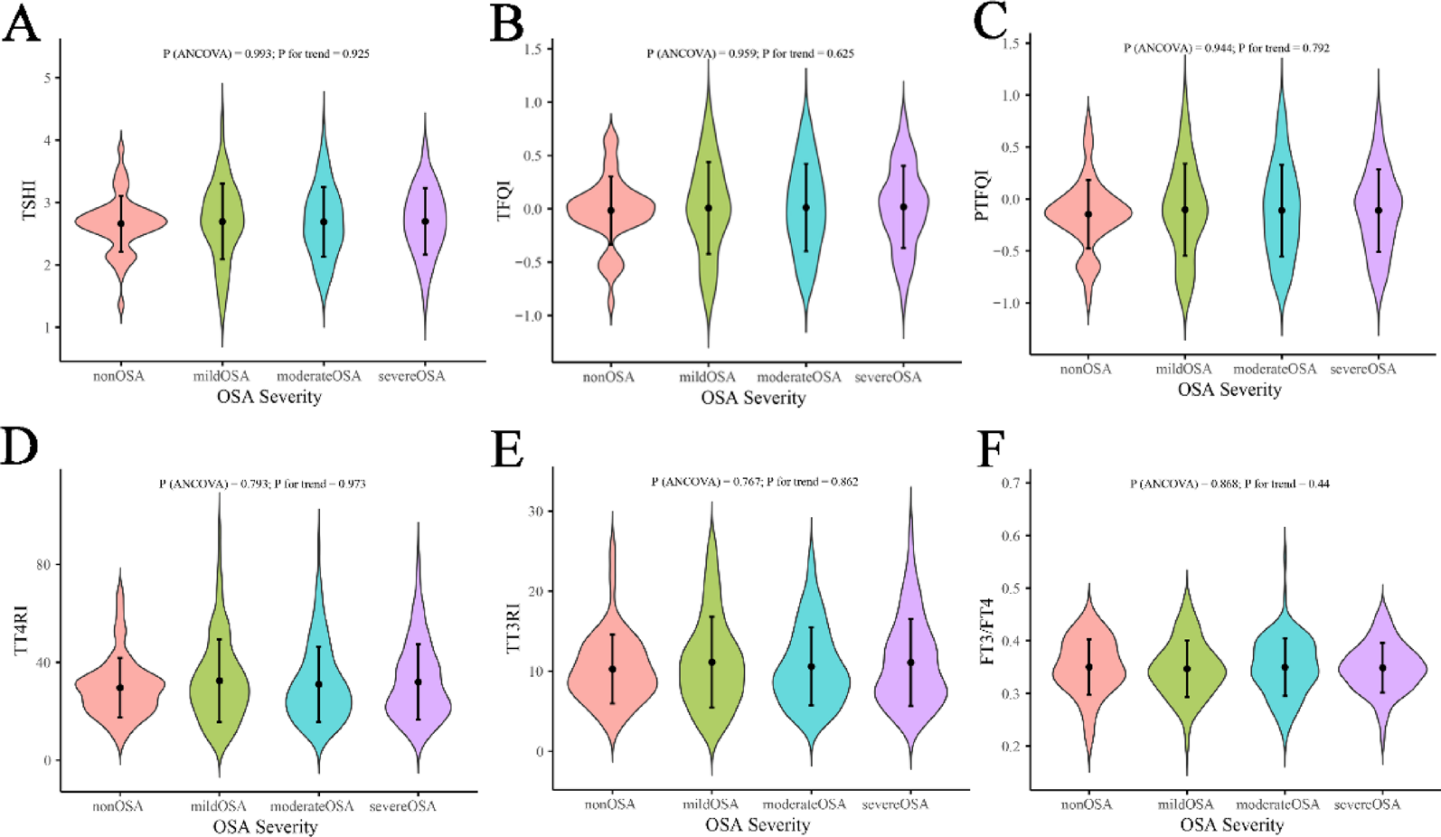
ANCOVA-based distribution of thyroid hormone sensitivity indices across OSA severity in males. ANCOVA, analysis of covariance; OSA, obstructive sleep apnea

After adjustment for age and BMI, analysis of covariance revealed significant linear trends across OSA severity categories in females but not in males. In females, TFQI (P for trend = 0.0395) and TT4RI (P for trend = 0.0293) increased progressively with OSA severity, whereas other indices, including PTFQI, TSHI, TT3RI, and the FT3/FT4 ratio, showed no significant trend (Fig. 1). In males, no thyroid hormone sensitivity index demonstrated a significant association with OSA severity (*P* > 0.05) (Fig. 2).

### Associations between OSA and thyroid hormone sensitivity indices

**Fig. 3:**
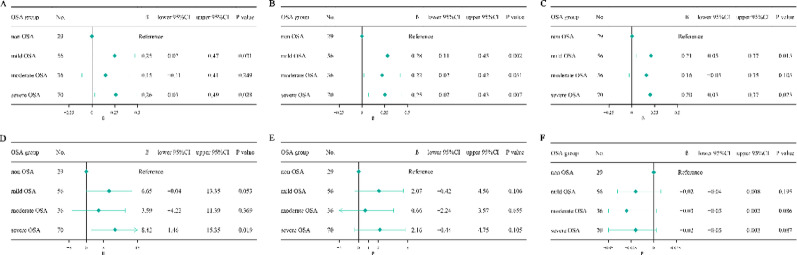
Regression-based changes in thyroid hormone sensitivity indices by OSA severity in females. (**a**) TSHI, (**b**) TFQI, (**c**) PTFQI, (**d**) TT4RI, (**e**) TT3RI, and (**f**) FT3/FT4 ratio. Abbreviations: OSA, obstructive sleep apnea; TSHI, TSH index; TFQI, thyroid feedback quantile-based index; PTFQI, parametric thyroid feedback quantile-based index; TT4RI, thyrotroph T4 resistance index; TT3RI, thyrotroph T3 resistance index

**Fig. 4:**
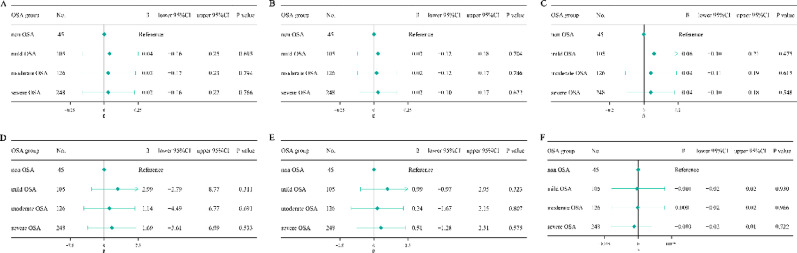
Regression-based changes in thyroid hormone sensitivity indices by OSA severity in males. (**a**) TSHI, (**b**) TFQI, (**c**) PTFQI, (**d**) TT4RI, (**e**) TT3RI, and (**f**) FT3/FT4 ratio. Abbreviations: OSA, obstructive sleep apnea; TSHI, TSH index; TFQI, thyroid feedback quantile-based index; PTFQI, parametric thyroid feedback quantile-based index; TT4RI, thyrotroph T4 resistance index; TT3RI, thyrotroph T3 resistance index

After adjustment for age, BMI, sleep duration, WASO, sleep efficiency, smoking, drinking, menopausal status (for female), hypertension, diabetes mellitus, and CHD, OSA was independently associated with higher TFQI (β = 0.26, 95% CI 0.10–0.42, *p* = 0.001), PTFQI (β = 0.20, 95% CI 0.05–0.35, *P* = 0.011), TSHI (β = 0.24, 95% CI 0.03–0.44, *P* = 0.025), and TT4RI (β = 6.82, 95% CI 0.59–13.05, *P* = 0.033) in females (Table 3). When stratified by OSA severity, a graded association was observed in females. Compared with the non-OSA group, mild (β = 0.28, 95% CI 0.11–0.45, *P* = 0.002), moderate (β = 0.22, 95% CI 0.02–0.42, *P* = 0.031), and severe OSA (β = 0.25, 95% CI 0.07–0.43, *P* = 0.007) were all associated with higher TFQI. Similarly, mild and severe OSA were significantly associated with increased PTFQI, TSHI, and severe OSA were significantly associated with increased TT4RI (*P* < 0.05). No meaningful associations were observed for TT3RI or FT3/FT4 ratio. In males, none of the thyroid hormone sensitivity indices showed any notable relationship with OSA or its severity (Figs. 3 and 4).

**Table 3:** Multivariate regression analysis of OSA and thyroid hormone sensitivity indices stratified by sex

Varibales	Male		Female	
	β (95% CI)	*P* value	β (95% CI)	*P* value
TFQI	0.03 (-0.10, 0.16)	0.650	0.26 (0.10, 0.42)	0.001
PTFQI	0.04 (-0.09, 0.18)	0.518	0.20 (0.05, 0.35)	0.011
TSHI	0.03 (-0.15, 0.21)	0.739	0.24 (0.03, 0.44)	0.025
TT4RI	1.83 (-3.27, 6.94)	0.482	6.82 (0.59, 13.05)	0.033
TT3RI	0.55 (-1.18, 2.28)	0.536	1.89 (-0.42, 4.21)	0.111
FT3/FT4 ratio	-0.002 (-0.02, 0.01)	0.821	-0.02 (-0.04, 0.003)	0.090

The model was adjusted for age, BMI, sleep duration, WASO, sleep efficiency, smoking, drinking, menopausal status (for female), hypertension, diabetes, and coronary heart disease

CI, confidence interval; OSA, obstructive sleep apnea; TFQI, thyroid feedback quantile-based index; TSHI, TSH Index; TT4RI, thyrotroph T4 resistance index; PTFQI, parametric thyroid feedback quantile–based index; TT3RI, thyrotroph T3 resistance index

### Nonlinear correlations between OSA-related parameters and thyroid hormone sensitivity indices stratified by sex

**Fig. 5:**
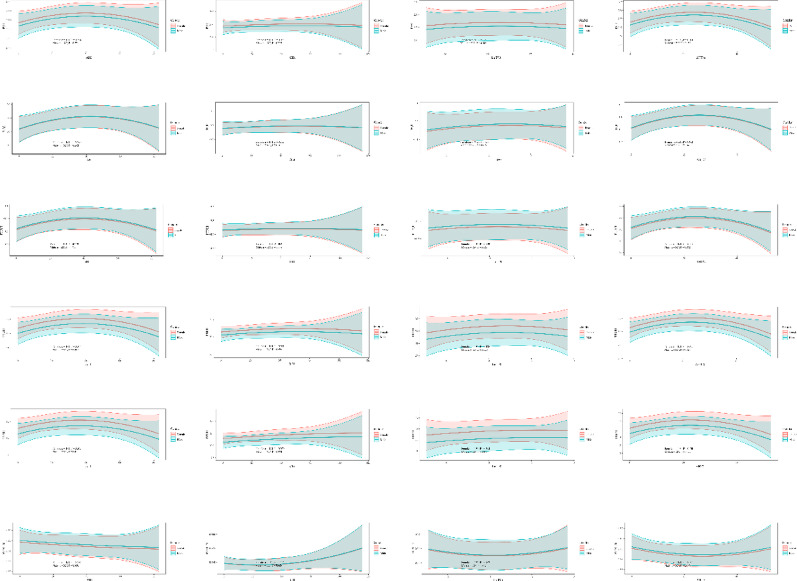
Quadratic prediction plots of thyroid hormone sensitivity indices by OSA-related parameters (AHI, ODI, LnT90, AHT%). Abbreviations: OSA, obstructive sleep apnea; AHI, apnea-hypopnea index; ODI, oxygen desaturation index; T90, percentage of time with oxygen saturation below 90%; AHT, apnea-hypopnea time; TSHI, TSH index; TFQI, thyroid feedback quantile-based index; PTFQI, parametric thyroid feedback quantile-based index; TT4RI, thyrotroph T4 resistance index; TT3RI, thyrotroph T3 resistance index

In females, AHI (*P* = 0.034), ODI (*P* = 0.021) were significantly correlated with TT4RI after adjustment for age, BMI, sleep duration, WASO, sleep efficiency, smoking, drinking and medical history. The fitted curves revealed a nonlinear pattern, where TSHI, TFQI, PTFQI, TT4RI, and TT3RI initially increased with higher OSA burden but tended to decline at the extreme end of AHI, ODI, or AHT%. Conversely, the FT3/FT4 ratio demonstrated an initial decrease followed by a slight rebound at higher OSA severity. In males, none of the associations reached statistical significance (Fig. 5).

## Discussion

In a well-characterized cohort of euthyroid adults, we observed that OSA was associated with impaired thyroid hormone sensitivity exclusively in females. Females with OSA demonstrated higher FT4, elevated central feedback indices (TFQI, PTFQI, TSHI, TT4RI), and a lower FT3/FT4 ratio, while no comparable alterations were present in males. The progressive increases in TFQI and TT4RI across OSA severity, together with the independent associations of OSA with higher TFQI, PTFQI, TSHI, and TT4RI after multivariable adjustment. Furthermore, AHI, ODI correlated with TT4RI in females, and central thyroid hormone sensitivity indices followed by attenuation at extreme OSA burden, with inverse behavior in FT3/FT4. These findings indicate that, in euthyroid females, OSA is linked to both reduced peripheral thyroid hormone sensitivity and impaired central thyroid hormone sensitivity, suggesting latent endocrine vulnerability prior to overt thyroid dysfunction.

The relationship between OSA and thyroid function has been contentious, with heterogeneous evidence regarding whether OSA is associated with higher or lower TSH and whether free thyroid hormones are increased or decreased. Clinical studies have shown that individuals with poor sleep quality tend to have elevated T4 and TSH [20];Patients with OSA, particularly those with coexisting obesity, are more susceptible to subclinical hypothyroidism [21]༛Imaging and functional studies have also revealed morphological changes in the thyroid gland among OSA patients, with linear correlations observed between AHI, SpO₂, and thyroid parameters [22]༛However, meta-analyses have reported a relatively low prevalence of thyroid dysfunction in individuals with OSA and no significant association between thyroid hormone levels and AHI [13]༛This inconsistency underscores a critical limitation of relying on absolute hormone levels to infer HPT-axis status: single-point FT4/FT3/TSH measurements capture concentration but not feedback dynamics. In contrast, thyroid hormone sensitivity indices reflect the responsiveness of thyrotrophs and the feedback setpoint of the HPT axis, and have demonstrated robust clinical relevance across metabolic phenotypes in euthyroid populations [11, 23–25].

Sleep apnea may contribute to hypothalamic inflammation [26]. Chronic intermittent hypoxia (CIH), a hallmark pathological feature of OSA, leads to elevated levels of reactive oxygen species (ROS), which activate multiple inflammatory mediators (IL-1β, IL-6, IL-8, TNF-α, and NF-κB)-that participate in neuroinflammatory processes and exacerbate neuronal damage [27]. These inflammatory cascades can disrupt central neurotransmitter signaling, hypothalamic-pituitary axis, and impair peripheral endocrine gland secretion and circadian rhythmicity [28]. Hypoxia and systemic inflammation can upregulate type 3 deiodinase (D3), which converts active T3 into inactive reverse T3, thereby reducing intracellular T3 levels [29, 30]. Deiodinase activity is regulated by ROS. Although IL-6 can enhance deiodinase expression, the oxidative stress it induces suppresses the activity of type 1 and 2 deiodinases (D1 and D2), reducing T3 production. Glutathione and thioredoxin, cofactors for deiodinase function, are depleted under oxidative stress, impairing the conversion of T4 to T3 [31].

Previous research has demonstrated that sex hormones exert significant regulatory effects on D1 activity. Compared to males, estrogen has a weaker modulatory effect on D1, which may lead to reduced T3 production in females [32]. This provides a plausible mechanistic basis for our observed decrease in the FT3/FT4 ratio and the corresponding increase in TT4RI. Further studies have demonstrated that the inflammatory and stress responses induced by CIH exhibit sex-specific differences [33]. These differences may lead to sex-dependent alterations in deiodinase regulation and in the feedback set-point of the HPT axis potentially explaining why females are more prone to central and peripheral thyroid hormone sensitivity abnormalities under increased OSA burden. Moreover, estrogen enhances the expression of thyrotropin-releasing hormone (TRH) receptors in the pituitary, rendering female pituitary cells more sensitive to TRH stimulation. Under chronic stress conditions, females may even exhibit elevated TRH expression [34]. In hypoxic or ischemic states, hypoxia-inducible factor 1-alpha can directly induce DIO3 gene expression, leading to a rapid increase in D3 activity and the establishment of a localized low-T3 state. In response to inflammatory injury, D2 expression may be upregulated to compensate for T3 deficiency [30, 35]. It is possible that the temporal dynamics of deiodinase expression contribute to the nonlinear biological effects of thyroid hormones. Early stimulation may provoke a hypothalamic-pituitary “hyper-response,” characterized by a rapid decline in circulating T3 levels and a compensatory rise in TSH. In contrast, during prolonged disease states, pituitary secretory rhythms and release capacity may become suppressed, resulting in reduced TSH output [36], This may explain the observed decline in TSH-based central thyroid hormone sensitivity indices under extreme OSA burden.

Our findings have important clinical implications. Thyroid hormone sensitivity indices may serve as early biomarkers of endocrine vulnerability in OSA patients with normal biochemical profiles, enabling more refined risk stratification and potentially guiding early intervention. Given the established associations between impaired thyroid hormone sensitivity and adverse metabolic outcomes, including insulin resistance, dyslipidemia, and increased cardiovascular risk, treating OSA may help restore HPT axis sensitivity and thereby mitigate downstream endocrine and metabolic complications. Moreover, the observed sex specificity highlights the importance of individualized endocrine assessment in sleep medicine. The incorporation of thyroid hormone sensitivity indicators into clinical practice can enhance the detection of subclinical dysfunction, provide a basis for treatment decisions, and ultimately improve the long-term prognosis of OSA patients. Nonetheless, several limitations should be acknowledged. The cross-sectional design limits causal inference, and longitudinal studies are needed to determine whether OSA treatment can reverse thyroid hormone sensitivity impairment. Although multiple indices were assessed, direct measurements of central regulators such as TRH were unavailable. Finally, sex-stratified associations need to be further validated in larger and more diverse populations to confirm biological relevance.

## Conclusions

Obstructive sleep apnea is associated with impaired thyroid hormone sensitivity in euthyroid adults, with significant alterations observed in females but not in males.

## Supplementary Information

Supplementary Material 1

## Data Availability

The datasets used and analyzed during the current study are available from the corresponding author on reasonable request.

## Abbreviations

OSA: obstructive sleep apnea
HPT: hypothalamic-pituitary-thyroid
TSH: thyroid-stimulating hormone
TFQI: thyroid feedback quantile-based index
PTFQI: parametric thyroid feedback quantile-based index
TSHI: TSH Index
TT4RI: thyrotroph T4 resistance index
TT3RI: thyrotroph T3 resistance index
FT3: free triiodothyronine
FT4: free thyroxine
AHI: apnea-hypopnea index
ODI: oxygen desaturation index
T90: percentage of time with oxygen saturation below 90%
Mean SpO₂: mean blood oxygen saturation
Mini SpO₂: minimum blood oxygen saturation
WASO: wake after sleep onset
AHT: apnea-hypopnea time
IQR: interquartile ranges
ANCOVA: analysis of covariance
BMI: body mass index
CI: confidence intervals
CIH: chronic intermittent hypoxia
ROS: reactive oxygen species
D3: type 3 deiodinase
D1: type 1 deiodinases
TRH: thyrotropin-releasing hormone
